# Plasticity of Select Primary Afferent Projections to the Dorsal Horn after a Lumbosacral Ventral Root Avulsion Injury and Root Replantation in Rats

**DOI:** 10.3389/fneur.2017.00291

**Published:** 2017-07-31

**Authors:** Allison J. Bigbee, Mahnaz Akhavan, Leif A. Havton

**Affiliations:** ^1^Department of Neurology, David Geffen School of Medicine at UCLA, Los Angeles, CA, United States; ^2^Department of Molecular, Cell, and Developmental Biology, UCLA, Los Angeles, CA, United States; ^3^Department of Neurobiology, David Geffen School of Medicine at UCLA, Los Angeles, CA, United States

**Keywords:** cauda equine, VGLUT1, isolectin B4, calcitonin gene-related peptide, dorsal root ganglion

## Abstract

Injuries to the conus medullaris and cauda equina portions of the spinal cord result in neurological impairments, including paralysis, autonomic dysfunction, and pain. In experimental studies, earlier investigations have shown that a lumbosacral ventral root avulsion (VRA) injury results in allodynia, which may be ameliorated by surgical replantation of the avulsed ventral roots. Here, we investigated the long-term effects of an L6 + S1 VRA injury on the plasticity of three populations of afferent projections to the dorsal horn in rats. At 8 weeks after a unilateral L6 + S1 VRA injury, quantitative morphological studies of the adjacent L5 dorsal horn showed reduced immunoreactivity (IR) for the vesicular glutamate transporter, VGLUT1 and isolectin B4 (IB4) binding, whereas IR for calcitonin gene-related peptide (CGRP) was unchanged. The IR for VGLUT1 and CGRP as well as IB4 binding was at control levels in the L5 dorsal horn at 8 weeks following an acute surgical replantation of the avulsed L6 + S1 ventral roots. Quantitative morphological studies of the L5 dorsal root ganglia (DRGs) showed unchanged neuronal numbers for both the VRA and replanted series compared to shams. The portions of L5 DRG neurons expressing IR for VGLUT1 and CGRP, and IB4 binding were also the same between the VRA, replanted, and sham-operated groups. We conclude that the L5 dorsal horn shows selective plasticity for VGLUT1 and IB4 primary afferent projections after an L6 + S1 VRA injury and surgical repair.

## Introduction

Brachial plexus and lumbosacral nerve root injuries may result in a combination of paralysis of peripheral organs and sensory impairments, including the development of neuropathic pain (1, 2). Although injuries to peripheral nerves and dorsal roots, which carry sensory fibers, represent established causes of neuropathic pain, isolated ventral root transection injuries with degeneration of efferent motor fibers may represent another cause of hyperalgesia and allodynia in experimental models (3–5). An at-level neuropathic pain presentation with allodynia, but in the absence of hyperalgesia, may develop in response to a lumbosacral ventral root avulsion (VRA) injury in rats (6). Interestingly, acute surgical repair in the form of direct implantation of the avulsed lumbosacral ventral roots into the lateral funiculus of the spinal cord promotes regenerative growth by spinal cord neurons with extension of axons into the replanted roots, functional reinnervation of the lower urinary tract, and amelioration of neuropathic pain (6–8). However, information on the effects of the VRA injury and repair on afferent projections into the dorsal horn as a potential contributor to sensory plasticity has remained sparse.

The main objective of this investigation was to study the effects of a unilateral L6 and S1 VRA injury, with and without surgical replantation of the avulsed ventral roots into the spinal cord, on the expression of select markers for subsets of primary afferents in the L5 dorsal horn. This experimental model interrupts the ventral horn exit zone at the junction between the central and peripheral nervous systems, but the dorsal horn entry zone remains anatomically intact and allows primary afferents to enter the spinal cord. We investigated the immunohistochemical expression patterns for the vesicular glutamate transporter (VGLUT1) and calcitonin gene-related peptide (CGRP), as well as the histochemical binding patterns for the isolectin B4 (IB4). VGLUT1 immunoreactivity (IR) is detected primarily within Rexed’s laminae III and IV, and the medial portion of lamina V of the dorsal horn, and serves as a marker for cutaneous and muscle afferents (9, 10). CGRP IR is normally present in Rexed’s laminae I and II and indicates small and thinly myelinated or non-myelinated peptidergic primary afferents (11). Staining for IB4 is also detected primarily in Rexed’s laminae I and II and used to identify the presence of non-peptidergic primary afferents (12, 13). We first identified the labeling and staining patterns for VGLUT1, CGRP, and IB4 in the L5 dorsal horn, then examined the corresponding L5 dorsal root ganglion (DRG) using the same morphological markers to examine signs for intramedullary plasticity of afferent projections and neuronal counts. This morphological investigation represents a direct extension of prior studies on VRA-induced at-level neuropathic pain and its amelioration by a surgical root reimplantation procedure, and the spinal cord and DRG tissues used for the present study were obtained from the same animals as those providing behavioral and morphological data for our prior report (6).

## Materials and Methods

All animal procedures were approved by the Chancellor’s Animal Research Committee at UCLA and performed according to the standards established by the National Institutes of Health (NIH) Guide for Care and Use of Laboratory Animals. All efforts were made to minimize the numbers of animals needed for the study and any suffering associated with the procedures. Adult female Sprague-Dawley rats (Charles River Laboratories, Wilmington, MA, USA) were included in the studies (*n* = 25; 200–220 g body weight). The rats were divided into three groups: (1) rats undergoing a sham operation procedure of a lumbar laminectomy and opening of the dura mater (LAM, *n* = 13); (2) rats undergoing an L6 and S1 VRA injury (VRA, *n* = 7); and (3) rats undergoing an L6 and S1 VRA injury followed by an acute implantation of lesioned ventral roots into the lateral funiculus of the corresponding segments (VRI, *n* = 5).

### Surgical Procedures

Spine surgeries with ventral root procedures were performed according to established protocols (6, 7). In short, all subjects were maintained under a surgical plane of inhalational anesthesia (2% isoflurane). A midline incision was made over the lumbar spinous processes, and an L1–L3 hemi-laminectomy was performed with spinous processes left intact. The dura was opened, and the L6 and S1 ventral roots were identified and avulsed from the spinal cord surface using a fine jeweler’s forceps to apply traction along the normal course of the ventral roots. In animals of the VRI series, the tip of a scalpel blade was used to make two small longitudinal incisions along the lateral funiculus of the L6 and S1 spinal cord segments, and the avulsed ventral roots were gently implanted into the spinal cord white matter. The reimplanted roots were held in place within the longitudinal spinal cord incision, aided by the application of adjacent coagulating blood, but without the use of any sutures or tissue glue. In the LAM series, the dura was opened, the L6 and S1 ventral roots identified, gently manipulated, but not injured. A layer of Gelfoam^®^ was positioned over the exposed spinal cord, and a titanium mesh was attached over the laminectomy site, secured to the ligaments between the T11–T12 and L5–L6 spinous processes using a 6-0 silk suture, to provide added stabilization of the vertebral column and protect the exposed spinal cord segments against any direct pressure from the surrounding tissue (14). The paraspinous muscle and skin layers were closed separately, and all rats were allowed to recover from the procedure. Buprenex^®^ (0.2–0.5 mg/kg s.c.) was given every 12 h for 2 days to provide postoperative pain control. All animals were checked daily for overall health, and bladders were manually expressed as needed during the recovery phase until independent voiding function was present.

### Morphological Studies

At 8 weeks postoperatively, all animals received an overdose of sodium pentobarbital and were perfused intracardially with 0.1 M phosphate buffer followed by a 4% paraformaldehyde solution. The vertebral column was removed and the spinal cord dissected free to visualize individual lumbosacral DRGs and nerve roots. Only animals with an anatomically verified L6 + S1 ventral root lesion with or without root repair were included in the studies. For the repair series of animals, only rats with replanted ventral roots still attached at the surgical repair site were included in the study. The spinal cord and lumbosacral DRGs from each subject were post-fixed in the fixative solution overnight and rinsed in 0.1 M phosphate-buffered saline (PBS). The spinal cord and DRG tissues were cryoprotected in a 30% sucrose solution for 24 h, preserved in OCT compound (Sakura Finetek, Torrance, CA, USA), and stored in −80°C. Spinal cord tissues were cut at 30-µm thickness, and DRG tissues were cut at 10-µm thickness using a cryostat.

Established criteria for the rat lumbosacral cytoarchitecture were used to identify the location of the L5 spinal cord (15). Every fifth section of the L5 spinal cord segment or DRG was selected for morphological analysis, and a total of 4–8 sections were analyzed for each morphological marker. Adjacent sections were processed for the detection of CGRP, VGLUT1, and IB4. Although the L6 + S1 ventral roots were avulsed, the morphological studies were performed using the adjacent L5 segment in order to minimize involvement of injured ventral root afferents of the L6 + S1 segments and to allow direct comparisons with prior pain behavioral and morphological studies in the same animals (6).

**Figure 1:**
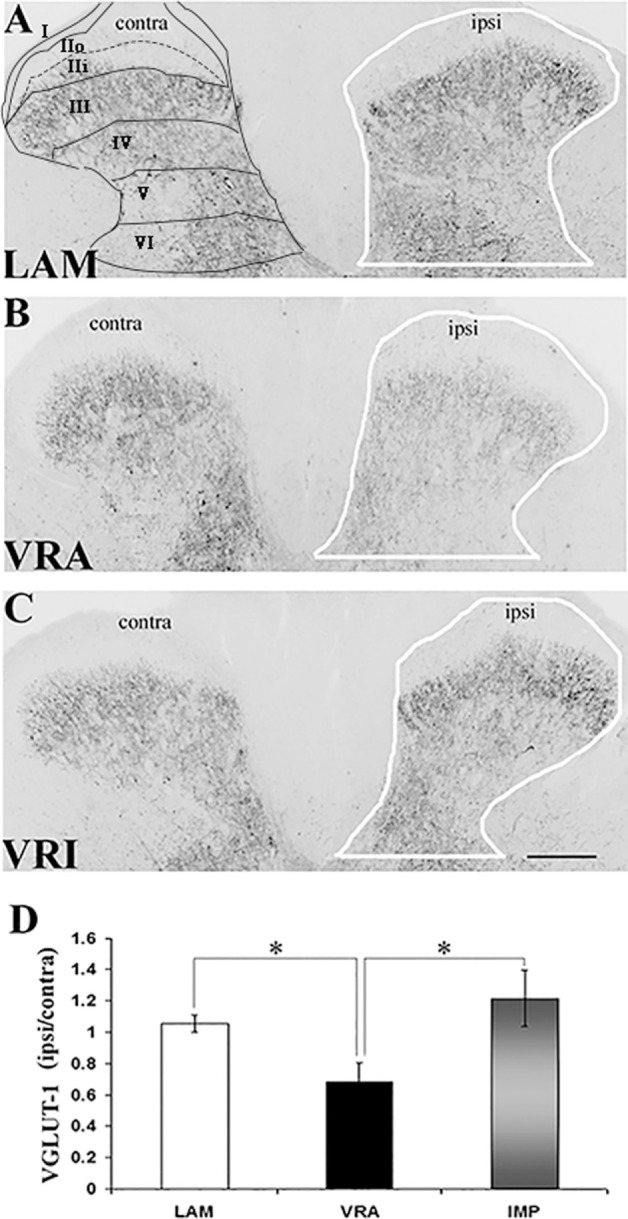
Effects of a unilateral L6 + S1 ventral root avulsion (VRA) injury and VRA injury followed by acute root replantation (VRI) on VGLUT1 immunoreactivity (IR) in the L5 dorsal horn at 8 weeks postoperatively. **(A–C)** The results were compared with sham-operated animals (LAM) and expressed as a ratio for the labeling detected on the sides ipsilateral (ipsi) and contralateral (contra) to the surgical procedures. The region of interest is indicated by the outline of the dorsal horn on the ipsi side for the LAM, VRA, and VRI groups. Rexed’s laminae of the dorsal horn are also indicated for LAM. **(D)** Note that VGLUT1 IR was decreased on the ipsi side after the VRA injury, whereas the corresponding VGLUT1 IR for the VRI group was symmetric and similar to the control group (LAM). Scale bar = 250 µm and * indicates a significant difference of *p* < 0.05 between groups.

**Figure 2:**
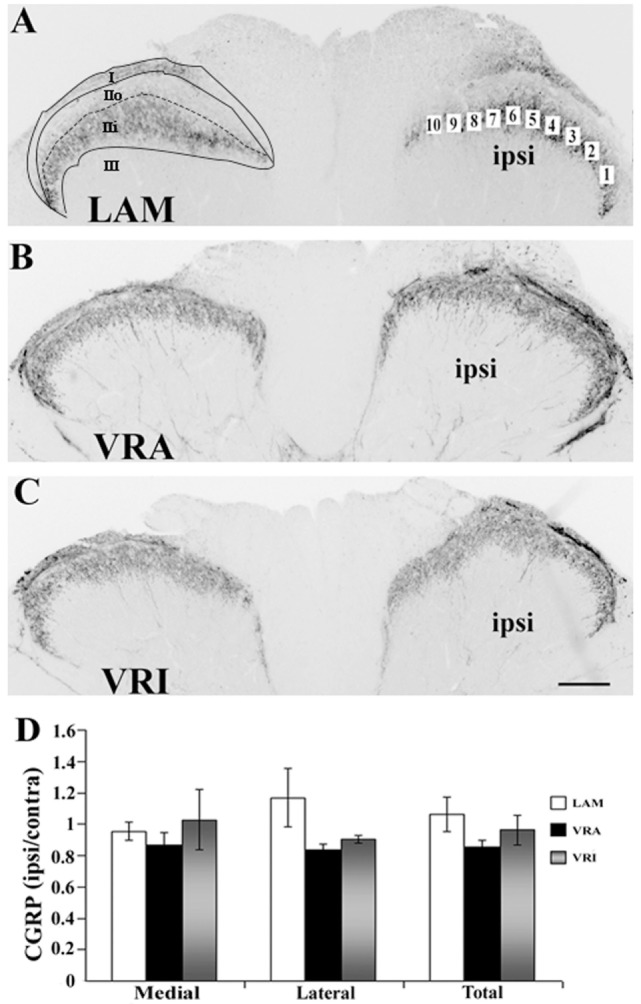
Effects of a unilateral L6 + S1 ventral root avulsion (VRA) injury and VRA injury followed by acute root replantation (VRI) on CGRP immunoreactivity (IR) in the L5 dorsal horn at 8 weeks postoperatively. **(A–C)** The results were compared with sham-operated animals (LAM) and expressed as a ratio for the labeling detected on the sides ipsilateral (ipsi) and contralateral (contra) to the surgical procedures. The region of interest was indicated by the placement of 10 boxes of 1,225 µm^2^ each across the superficial laminae of the dorsal horn for the LAM, VRA, and VRI groups. For comparisons between the lateral and medial portions of the dorsal horn, boxes 1–5 represented the lateral portion and boxes 6–10 represented the medial portion of the superficial laminae. Rexed’s laminae for the superficial dorsal horn are indicated for LAM. **(D)** Note that there was no difference between the LAM, VRA, and VRI groups with regards to CGRP IR in the superficial laminae of the dorsal horn. Scale bar = 250 µm.

**Figure 3:**
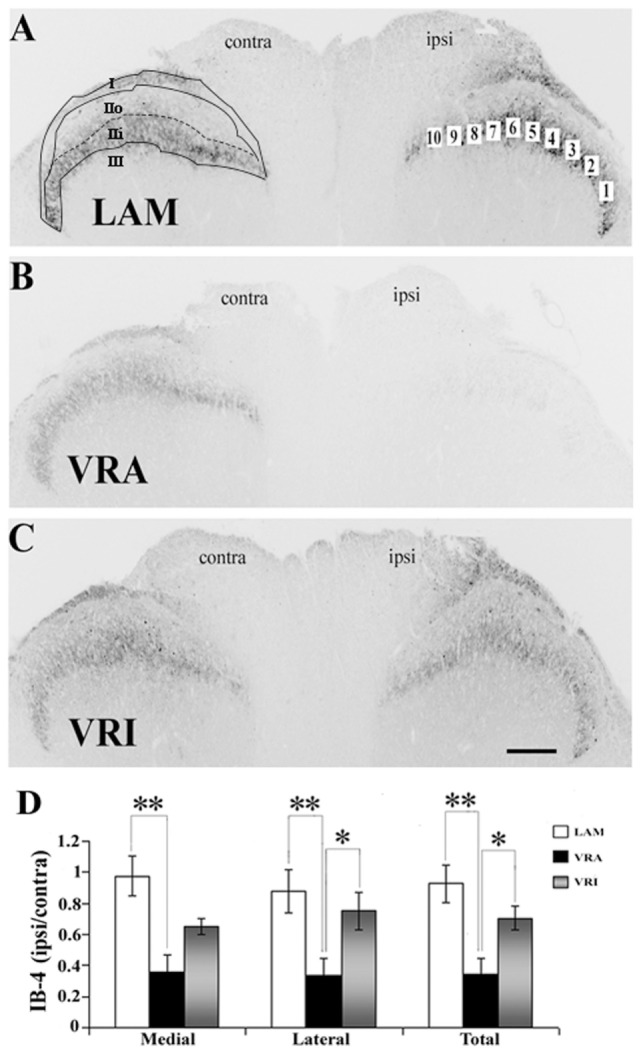
Effects of a unilateral L6 + S1 ventral root avulsion (VRA) injury and VRA injury followed by acute root replantation (VRI) on isolectin B4 (IB4) binding in the L5 dorsal horn at 8 weeks postoperatively. **(A–C)** The results were compared with sham-operated animals (LAM) and expressed as a ratio for the labeling detected on the sides ipsilateral (ipsi) and contralateral (contra) to the surgical procedures. The region of interest was indicated by the placement of 10 boxes of 1,225 µm^2^ each across the superficial laminae of the dorsal horn for the LAM, VRA, and VRI groups. For comparisons between the lateral and medial portions of the dorsal horn, boxes 1–5 represented the lateral portion and boxes 6–10 represented the medial portion of the superficial laminae. Rexed’s laminae for the superficial dorsal horn are indicated for LAM. **(D)** Note that there was a reduced IB4 binding on the ipsi side after the VRA injury, whereas the corresponding IB4 binding was symmetric and similar between the LAM and VRI groups. Scale bar = 250 µm and * indicates a significant difference of *p* < 0.05 and ** indicates a significant difference of *p* < 0.01 between groups.

Sections of the L5 spinal cord segment and the L5 DRGs were initially rinsed in PBS. Next, the sections were blocked in 5% normal donkey serum for 1 h (Jackson Immuno Research Labs, Inc., West Grove, PA, USA) and processed for VGLUT1 (anti-rabbit, 1:1,000; Synaptic Systems, Göttingen, Germany), CGRP (anti-rabbit, 1:4,000, Millipore, Billerica, MA, USA), or IB4 FITC-conjugated lectin (1:100, Sigma, St. Louis, MO, USA). The primary antibodies were incubated overnight in 0.3% Triton X-100 in PBS at room temperature. Secondary antibodies (Alexa Fluor^®^ 594 or Alexa Fluor^®^ 488, 1:500, Invitrogen, Carlsbad, CA, USA) were incubated for 1 h at room temperature. The sections underwent a final rinse and were mounted on glass slides with Vectashield mounting medium (Vector Laboratories, Burlingame, CA, USA). The region of interest (ROI) for the analysis of VGLUT1 IR consisted of an outline of the dorsal horn gray matter with its ventral border at the same level as the ventral tip of the dorsal columns (Figures 1A–C). The ROI for the analysis of CGRP IR and IB4 labeling consisted of a total of 10 square boxes with an area of 1,225 µm^2^ each across the inner portion of Rexed’s lamina II (Figures 2A and 3A). Fluorescent images were captured using a Spot camera (Diagnostic Instruments, Sterling Heights, MI, USA), which was attached to a Nikon E600 microscope. Magnification, light intensity, and exposure times were held constant during the image capturing. Densitometric analysis and quantitative studies were performed using C-Imaging software (Compix, Inc., Brandywine, PA, USA) and determined an area of immunopositive labeling above a constant threshold. ROIs included the dorsal horn gray matter for VGLUT1 IR studies and the superficial gray matter (laminae I–II) for CGRP IR and IB4 labeling. For quantitative studies of neuronal counts of the L5 DRGs, DAPI staining allowed for nuclear identification and for neuronal profile counts. Neuronal and non-neuronal nuclei showed distinct morphological differences with the neuronal nuclei being larger, round to oval in shape and showing less intense fluorescent labeling with uneven chromatin patterns, whereas glial nuclei showed small and round nuclei with bright fluorescent labeling. Each L5 DRG was cut serially at 10-µm thickness, and every fifth section was stained for DAPI and used to determine the total number of sensory neuron. Abercrombie’s method was applied to correct for the presence of split nuclei in sections using the formula, *N* = *A* × (*T*/(*D* + *T*)), wherein *N* is the corrected number of nuclei in a section, *A* is the crude count of nuclear profiles, *T* is the section thickness, and *D* is the average diameter of the nuclei (16). For each DRG, a total of 100 nuclei were measured to determine the average nuclear diameter. A total of 6–14 sections were used to determine the corrected neuronal count for each DRG. To calculate the proportion of sensory neurons labeled for VGLUT1, CGRP, IB4, and CGRP/IB4, profile counts for each of these subtypes of sensory neurons were determined and related to the corrected number of DAPI-stained DRG neurons in four sections from the mid portion of each DRG.

### Statistical Analyses

Data were expressed as mean ± SE. Analyses between experimental groups were performed using the non-parametric Kruskal–Wallis ANOVA with Dunn’s *post hoc* test. We considered *p* < 0.05 as statistically significant.

## Results

We investigated the effects of an L6 and S1 VRA injury and repair on the plasticity of primary sensory afferent projections to the dorsal horn of the L5 segment in rats. For this purpose, we examined the IR for the vesicular glutamate transporter, VGLUT1, and CGRP, as markers for the spinal cord projections of mechanoreceptive primary afferents and peptidergic nociceptive primary afferents, respectively. We also studied the binding of the Griffonia simplicifolia IB4, as a marker for non-peptidergic primary afferent projections to the dorsal horn.

### VGLUT1 IR in the Dorsal Horn

VGLUT1 IR was detected in the dorsal horn of the L5 segment in all groups, especially within laminae IV–VI. Using densitometry, IR for VGLUT1 was quantified in the dorsal horn (laminae I–VI) of both the ipsilateral (ipsi) and contralateral (contra) sides in rats of the LAM, VRA, and VRI groups (Figure 1). The VGLUT1 IR was markedly reduced in the dorsal horn after the VRA injury, as demonstrated by a decreased ratio of the ipsi to contra VGLUT1 IR in this ROI in rats of the VRA series (0.68 ± 0.13, *n* = 6) compared to the corresponding ratio in rats of the LAM series (1.05 ± 0.05, *n* = 8, *p* < 0.05). However, the ipsi to contra ratio of VGLUT1 IR was maintained in the VRI series (1.21 ± 0.18, *n* = 5) and not different from the corresponding ratio in rats of the LAM series but higher than the VGLUT1 IR in the VRA group (*p* < 0.05).

### CGRP IR and Isolectin IB4 Binding in the Dorsal Horn

Both CGRP IR and isolectin IB4 were primarily detected within lamina I and II of the superficial dorsal horn in rats of all series. Double labeling for CGRP IR and IB4 binding combined with confocal light microscopy demonstrated that their territories largely overlapped in the superficial dorsal horn. Specifically, CGRP IR was primarily detected in lamina I and lamina IIo, whereas isolectin IB4 binding was mostly detected in lamina IIo and lamina IIi. There was no or minimal co-localization of CGRP IR and isolectin IB4 binding in the dorsal horn. Densitometry showed that the ratio of ipsi to contra values for CGRP in the superficial dorsal horn was not different between the LAM, VRA, and VRI groups (Figure 2).

The binding of IB4 was in the same ROI markedly reduced after the VRA injury, as demonstrated by a decreased ratio of ipsi to contra binding of IB4 in the superficial dorsal horn (0.35 ± 0.10, *n* = 7) compared to the corresponding ratio in rats of the LAM series (0.93 ± 0.12, *n* = 8, *p* < 0.05) (Figure 3). Interestingly, the ratio of ipsi to contra binding for binding for IB4 in the superficial dorsal horn was maintained in the VRI series (0.71 ± 0.08, *n* = 5) and not different from corresponding binding in the LAM series but significantly higher than IB4 binding ratio in the rats of the VRA series (*p* < 0.05) (Figure 3). When the medial and lateral portions of the superficial dorsal horn were analyzed separately, similar statistically significant differences between the experimental groups were identified.

### Neuronal Numbers Functional Phenotypes in the L5 DRG

**Figure 4:**
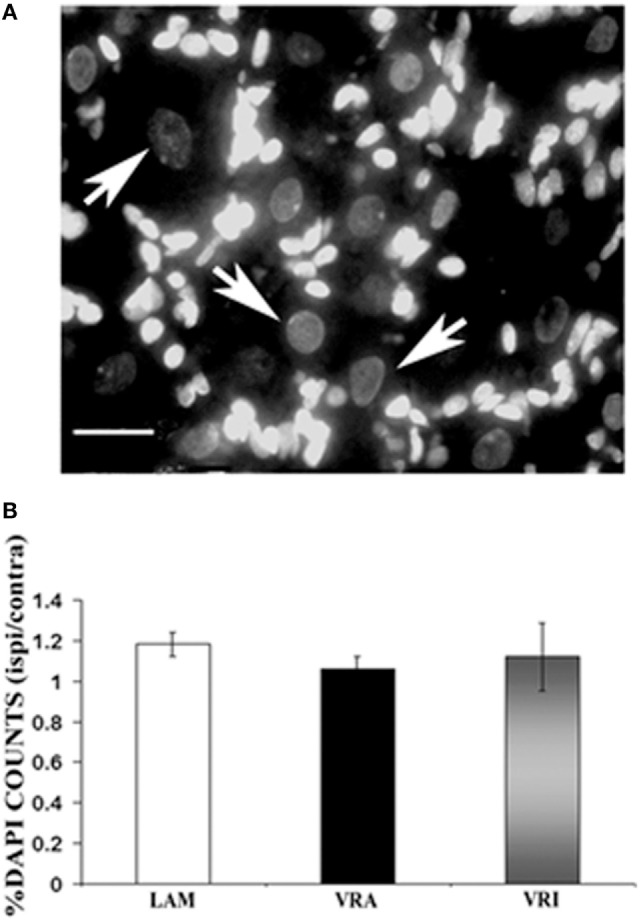
Effects of a unilateral L6 + S1 ventral root avulsion (VRA) injury and VRA injury followed by acute root replantation (VRI) on neuronal counts in the L5 dorsal root ganglion (DRG) at 8 weeks postoperatively. The results were compared with sham-operated animals (LAM) and expressed as a ratio for the labeling detected on the sides ipsilateral (ipsi) and contralateral (contra) to the surgical procedures. **(A)** DAPI staining allowed for the identification and counting of neuronal nuclei in the L5 DRG (arrows). **(B)** Note that there was no difference with regards to neuronal counts of the L5 DRG between the LAM, VRA, and VRI groups. Scale bar = 20 µm.

The effects of an L6 + S1 VRA or VRI on the L5 DRG were determined by quantitative light microscopy. The ratio of DAPI-stained DRG neurons of the ipsi and contra L5 DRG was determined for subjects of the LAM, VRA, and VRI series (Figure 4). The ipsi/contra ratio for the L5 DRG was 1.00 ± 0.07 (*n* = 5), 1.00 ± 0.04 (*n* = 5), and 1.09 ± 0.06 (*n* = 5), respectively. There were no differences between the groups.

**Figure 5:**
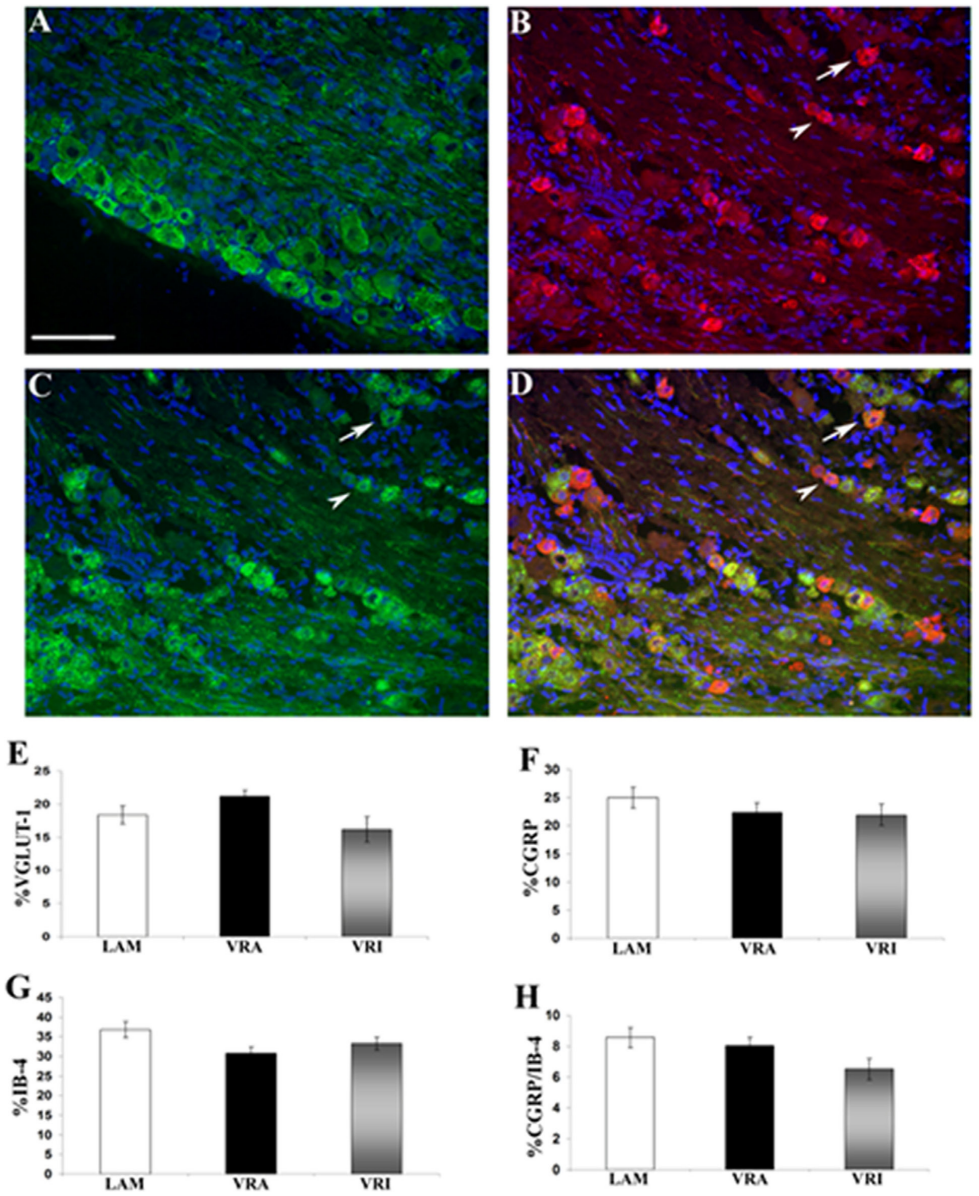
Effects of a unilateral L6 + S1 ventral root avulsion (VRA) injury and VRA injury followed by acute root replantation (VRI) on the relative proportions of neurons showing immunoreactivity (IR) for **(A)** VGLUT1 or **(B)** calcitonin gene-related peptide (CGRP) or **(C)** binding for isolectin B4 (IB4) in the L5 dorsal horn at 8 weeks postoperatively. The results were compared with sham-operated animals (LAM) and expressed as a ratio for the labeling detected on the sides ipsilateral (ipsi) and contralateral (contra) to the surgical procedures. Note that there was no difference with regards to the percent dorsal root ganglion (DRG) neurons expressing IR for **(E)** VGLUT1 or **(F)** GCRP or **(G)** binding for IB4 between the LAM, VRA, and VRI groups. **(H)** In addition, there was no difference for the number of DRG neurons showing double labeling for CGRP and IB4 between the groups. Scale bar = 100 µm.

The relative frequency for the expression of VGLUT1 IR, CGRP IR, and binding for IB4 was determined for the ipsi L5 DRG neurons of the LAM, VRA, and VRI series (Figure 5). No statistical differences were detected for the relative frequencies for VGLUT1 IR, CGRP IR, or IB4 binding between the groups. In addition, there was no difference between the experimental groups with regards to the frequency of co-labeling of CGRP IR and IB4 binding.

## Discussion

The present study demonstrated differential plasticity for the expression of VGLUT1, IB4, and CGRP in the dorsal horn of the L5 spinal cord segment at 8 weeks after a combined L6 + S1 VRA injury as well as after a combined L6 + S1 VRA injury followed by acute replantation of the avulsed ventral roots. Specifically, the expression of VGLUT1 was reduced in the deep dorsal horn at 8 weeks after the VRA. In the superficial dorsal horn, the expression of IB4 was also reduced, whereas CGRP IR remained unchanged after the VRA injury. When the lumbosacral VRA injury was followed by an acute implantation of the lesioned roots into the lateral funiculus of the affected spinal cord segments, the L5 dorsal exhibited baseline expression levels for VGLUT1, IB4, and CGRP, suggestive of a restoration or preservation of primary afferent phenotype expressions.

### Plasticity of Primary Sensory Afferents after Peripheral Injury

It is well established that injuries to the peripheral nervous system, including a spinal nerve or sciatic nerve ligation, may result in neuropathic pain and plasticity of the intramedullary primary afferent projections (17–20). Prior studies have demonstrated a reduction of both VGLUT1 and isolectin IB4 in the lumbar dorsal horn after a sciatic nerve lesion (21, 22) and decreased CGRP and isolectin IB4 binding in the L4 and L5 dorsal horns after an L5 spinal nerve ligation (23). Such injuries to the peripheral nervous system may also result in the degeneration and death of axotomized DRG neurons (13, 24, 25). It is therefore possible that a loss of axotomized DRG neurons may have contributed to the reduced intramedullary labeling of markers for primary afferents after a mixed nerve or spinal nerve root lesion. In contrast, a VRA injury is sensory-sparing, and no DRG loss or change in the relative numbers of VGLUT1, CGRP, or IB4 positive neurons was detected in the L5 DRG after an L6 + S1 VRA injury. Our observed downregulation of VGLUT1 IR and isolectin IB4 staining in the L5 dorsal horn in the VRA series is therefore likely a representation of plasticity within select primary afferent projections.

In addition to the at-level injury and effects seen in the present study at the L5 segment after an L6 + S1 VRA injury and repair, plasticity may also take place at more remote spinal segments, which are involved with autonomic functions. The lower urinary tract is under parasympathetic control by autonomic innervation that originates primarily from the L6 and S1 segments, and it receives sympathetic innervation with origin at the lower thoracic and upper lumbar segments (26, 27). Both CGRP and VGLUT1 are present in distinct autonomic fibers that innervate the dorsal horn and autonomic nuclei at the thoracolumbar and lumbosacral segments (28–31). Following an L6 + S1 VRA injury, there was a selective decrease of CGRP in the dorsal horn of the L1 + L2 dorsal horn and no detectable effect on the VGLUT1 innervation of the same segments at 8 weeks postoperatively, whereas an acute reimplantation of the avulsed ventral root resulted in a partial restoration of the CGRP levels in the L1 + L2 dorsal horn (32). We conclude that the L6 + S1 VRA injury and repair may have parallel but different effects on spinal cord innervation by both somatosensory and visceral afferents.

### Neuroprotective Effects of Avulsed Ventral Root Reimplantation

A neuroprotective effect provided by acute reimplantation of avulsed lumbosacral ventral roots is well established. Surgical reimplantation of avulsed L6 and S1 ventral roots into the lateral funiculus of the rat spinal cord augments motoneuron survival and regeneration of axons into the grafted roots (7), promotes reinnervation of peripheral targets (8), ameliorates neuropathic pain and intramedullary inflammatory and glial reactions in the dorsal horn (6), and reduces intramedullary degeneration of primary afferent axon collaterals in the spinal dorsal columns (33). In the present study, surgical reimplantation of avulsed L6 + S1 ventral roots resulted in the preservation of the intramedullary phenotype of VGLUT1 and CGRP IR and IB4 staining in the L5 dorsal horn. The latter finding represents a previously not known outcome of this surgical repair procedure.

The mechanisms behind the neuroprotective effects provided by the surgical reimplantation of avulsed ventral roots are not well understood. For the present study, it is unclear whether the surgical reimplantation of avulsed ventral roots restored a reduced expression of select primary afferent markers or protected primary afferent projections against structural degeneration. A downregulation of markers associated with transmitter function in the spinal cord is possible after a peripheral injury. Specifically, myelinated primary afferents to laminae III, IV, and IX in the rat spinal cord showed depletion of VGLUT1 IR after a sciatic nerve transection injury (22). Future studies are needed to clarify the effects of VRA injury on the integrity and the mechanisms for the protection of primary afferent marker expressions by root reimplantation.

### Functional Aspects

Our morphological findings may be compared with prior pain behavioral studies of sensory thresholds after a lumbosacral VRA injury and ventral root reimplantation in rats. Specifically, the examined spinal cord tissues were obtained from the same animals that developed long-term allodynia in the absence of hyperalgesia within the L5 dermatome after an L6 + S1 VRA injury (6). It is therefore interesting to note that the long-term development of neuropathic pain is associated with decreased IR for VGLUT1 and reduced staining for isolectin IB4 in the L5 dorsal horn, whereas there is normal IR for CGRP, at 8 weeks after a combined L6 + S1 VRA injury. Interestingly, the animals in the surgical treatment group showed gradual amelioration of the neuropathic pain within the L5 dermatome when the VRA injury was followed by an acute ventral root reimplantation (6). The absence of neuropathic pain at 8 weeks postoperatively was associated with normal labeling and staining patterns for VGLUT1, IB4, and CGRP in the L5 dorsal horn. In addition, a VRA injury-associated degeneration of primary afferent collaterals in the spinal dorsal columns was also ameliorated by reimplantation of avulsed ventral roots into the spinal cord of the same rats (33). We conclude that the plasticity for select primary afferent markers in the L5 dorsal horn was closely associated with our previously reported state of allodynia and the normalization of sensory threshold in the VRA injury and surgical root reimplantation groups, respectively.

## Ethics Statement

This study was carried out in accordance with the recommendations of the National Institutes of Health (NIH) Guide for Care and Use of Laboratory Animals and the Chancellor’s Animal Research Committee at UCLA. The protocol was approved by the Chancellor’s Animal Research Committee at UCLA.

## Author Contributions

AB and LH designed the study; AB and MA performed experiments; AB, MA, and LH interpreted the data; LH supervised the studies and wrote the manuscript. All authors reviewed and edited the manuscript.

## Conflict of Interest Statement

The authors declare that the research was conducted in the absence of any commercial or financial relationships that could be construed as a potential conflict of interest.
